# Sucrose synthase gene *SUS3* could enhance cold tolerance in tomato

**DOI:** 10.3389/fpls.2023.1324401

**Published:** 2024-01-25

**Authors:** Shouming Li, Ying Wang, Yuanyuan Liu, Changhao Liu, Wei Xu, Yongen Lu, Zhibiao Ye

**Affiliations:** ^1^ Key Laboratory of Special Fruits and Vegetables Cultivation Physiology and Germplasm Resources Utilization (Xinjiang Production and Construction Crops), College of Agriculture, Shihezi University, Shihezi, China; ^2^ National Key Laboratory for Germplasm Innovation and Utilization of Horticultural Crops, Huazhong Agricultural University, Wuhan, China; ^3^ Facility Horticulture Research Institute, Shihezi Academy of Agriculture Science, Shihezi, China; ^4^ Vegetable Research Institute, Wuhan Academy of Agricultural Sciences, Wuhan, China

**Keywords:** tomato, sucrose synthase, SUS3, cold stress, transcriptome

## Abstract

Tomatoes are susceptible to damage from cold temperatures in all stages of growth. Therefore, it is important to identify genetic resources and genes that can enhance tomato’s ability to tolerate cold. In this study, a population of 223 tomato accessions was used to identify the sensitivity or tolerance of plants to cold stress. Transcriptome analysis of these accessions revealed that *SUS3*, a member of the sucrose synthase gene family, was induced by cold stress. We further investigated the role of *SUS3* in cold stress by overexpression (OE) and RNA interference (RNAi). Compared with the wild type, *SUS3*-OE lines accumulated less MDA and electrolyte leakage and more proline and soluble sugar, maintained higher activities of SOD and CAT, reduced superoxide radicals, and suffered less membrane damage under cold. Thus, our findings indicate that *SUS3* plays a crucial role in the response to cold stress. This study indicates that *SUS3* may serve as a direct target for genetic engineering and improvement projects, which aim to augment the cold tolerance of tomato crops.

## Introduction

Tomato (*Solanum lycopersicum*) is an economically significant vegetable crop globally, originating from tropical and subtropical regions. It is sensitive to low temperatures, which adversely affects tomato growth, development, and ripening (Foolad and Lin, 2001; Venema et al., 2005). Cold stress has become the primary threat to early spring cultivation of tomatoes in Xinjiang, leading to substantial losses for farmers. Therefore, it is significant and valuable to breed modern cold-tolerant varieties in tomato, and it is essential for us to exploit resistant germplasm resources and to further understand the physiological and biochemical processes that promote cold tolerance.

Previous studies on plant cold acclimatization have shown that enzymes involved in the metabolism of antioxidants, lipids, carbohydrates, proline, and polyamines, as well as cold resistance-related proteins, and other osmotic regulation-related proteins, play an important role in plant resistance to cold stress (Li et al., 2011; Sato et al., 2011; Wang et al., 2011; Fan et al., 2012). Moreover, the overexpression of particular genes in pepper (Airaki et al., 2012), grape (Sawicki et al., 2012), and *Arabidopsis* (Hannah et al., 2005) can significantly improve the transgenic plants’ ability to tolerate cold, drought, and osmotic stresses.

Sucrose synthase (SUS) was first discovered in wheat germ by Cardini et al. (1955), and later, Geigenberger and Stitt (1993) demonstrated its role in regulating various metabolic pathways involving sucrose in plants. The SUS is a multigene family, with each family consisting of at least two gene members. In some species, more SUS families have been found. The tomato SUS family, for example, consists of six genes, namely, *SUS1*, *SUS3*, *SUS4*, *SUS5*, *SUS6*, and *SUS7*, distributed across five chromosomes (Bieniawska et al., 2007). In addition, grape and sugarcane have five SUS family genes identified (Zhang et al., 2013), and more than six have been found in cotton, *Arabidopsis*, and rice (Tatsuro et al., 2008; Chen et al., 2012; Zou et al., 2013).

The expression of SUS can be prompted by environmental factors, including low oxygen, low or high temperatures, and drought. In tomato roots under low oxygen stress, the overall capacity for sucrose hydrolysis remains unaltered, suggesting that SUS is adopted by tomato to enhance energy utilization efficiency and conserve ATP under hypoxic conditions. The SUS pathway can compensate, at least to some extent, for the inadequate energy supply caused by hypoxia (Sasaki et al., 2001). In barley, the SUS genes exhibit distinct responses to various abiotic stresses. For instance, hypoxia stress can induce *HvSUS1*, while drought can induce *HvSUS3* (Ricard et al., 1998). *HbSUS5* expression was found to be enhanced in the roots and leaves of rubber trees (*Hevea brasiliensis*) in response to low temperature and drought, indicating a significant role in stress response (Xiao et al., 2014). During the maturation of rice seeds, exposure to high temperatures may lead to the occurrence of chalkiness in brown rice. The overexpression of *SUS3* provides resistance to chalkiness in brown rice (Takehara et al., 2018).

A previous study showed that the SUS has been identified in many plants, such as tomatoes, tobacco, grape, rice, and *Arabidopsis*. However, the SUS gene regulation mechanism and biological function and the function of the *SUS3* gene underlying cold tolerance have not been further studied. Our study found that *SUS3* was highly expressed in the leaves and was induced under cold stress. Overexpression of *SUS3* improved its ability in resisting low temperatures during the seedling stage, indicating that *SUS3* plays an important role in tomato low-temperature resistance. Our research provides new insight into the biological function of *SUS3* and reveals a new gene that contributes to the cold tolerance of tomato.

## Materials and methods

### Tomato cold tolerance identification

Two hundred and twenty-three tomato accessions, consisting of 2 *Solanum cheesmaniae* (wild), 16 *S. pimpinellifolium* (PIM), 65 *S. lycopersicum* var. *cerasiforme* (CER), 105 *S. lycopersicum* (BIG), and 35 Guangxi accessions (**Supplementary Table S1**), were collected by our laboratory at Wuhan National Key Laboratory for Germplasm Innovation and Utilization of Horticultural Crops, China, in spring 2018. Four-week-old seedlings were placed in a constant temperature room at 4°C. After 7 days, the symptoms of cold damage on the plants were observed and the images were recorded. Cold damage was graded to five levels: level 0 indicated no symptoms and no sensitivity to the current low temperature; level 1 was characterized by injury spots on the edge of true leaves; level 2 was characterized by large-area injury spots on true leaves; level 3 was characterized by large-area injury spots on true leaves with leaf curling; and level 4 was characterized by wilting of plants and even necrosis of apical meristem (Wang et al., 1996). Three biological replicates were analyzed, and each replicate consisted of 15 individual plants.

### RNA sequencing analysis

Four-week-old seedlings were subjected to cold stress treatments at 4°C for 48 h. Twenty cold-sensitive and 20 cold-tolerant seedlings were selected. After treatment, the third fully expanded leaf from the top of each seedling was collected respectively, frozen in liquid nitrogen, and then stored at −80°C. The leaves were sent to Beijing Novogene Technology Co., Ltd. (Wuhan Branch) for library construction and sequencing. We used the average FPKM (expected number of fragments per kilobase of transcript sequence per million base pairs sequenced) value as a measure of gene expression.

### Abiotic stress tolerance assays

The cold-tolerant A57 (*S. lycopersicum* cv. Ailsa Craig) was used to study the expression of *SUS3*. Four-week-old seedlings were subjected to abiotic stress treatments, namely, ABA, ethylene, drought, cold, and salt stress treatments. The methods corresponding to these abiotic stress treatments were described previously (Song et al., 2016). The soil on the surface of the plants was washed off, and the residual water on the surface was absorbed. The plants were divided into five groups for different treatments. Plants in group 1 were placed in a dry environment at room temperature to induce drought stress. The roots of plants in group 2 were immersed in 400 mM of NaCl solution to induce salt stress; plants in group 3 were placed in a 4°C cold chamber to induce cold stress. The plants in group 4 and group 5 were sprayed with 100 μM of ABA and 200 μM of ethylene solutions for hormone induction treatment, respectively. Then, 0, 1, 3, 6, 12, and 24 h after each treatment, the second leaf from the apical meristem was collected, snap-frozen in liquid nitrogen, and stored at −80°C for RNA extraction.

### RNA extraction and gene expression analysis

Total RNA was extracted using the TRIzol reagent (Invitrogen, USA) and further reverse-transcribed into cDNA using a HiScript II 1st Strand cDNA Synthesis Kit (Vazyme, China). The relative transcript levels of specific genes were quantified using real-time PCR (qRT-PCR), which was conducted using a QuantStudio™ 6 Flex System (ABI, USA). The expression level of the *actin* gene (*Solyc11g005330*) was used as an internal control. For each biological replicate, the relative expression level of *SUS3* was quantified using three technical replicates and calculated using the 2^−ΔΔCT^ method. The related primer sequences can be found in **Supplementary Table S2**.

### Vector construction and genetic transformation

Cold-sensitive M82 (*S. lycopersicum*) was selected for transgenic tomato plants. For overexpression constructs, the complete open reading frame (ORF) of *SUS3* was amplified from M82 using *SUS3*-OE primers (**Supplementary Table S2**), and these constructs were cloned into pHELLSGATE8 vectors. An RNA interference (RNAi) construct, a 412-bp fragment from the *SUS3* gene, was amplified from cDNA that was prepared from M82 using the *SUS3*-Ri primers (**Supplementary Table S2**) and cloned into pHGRV using Clonase BP (Invitrogen, USA). M82 was transformed with all of the recombinant plasmids using *Agrobacterium* (strain *GV3101*)-mediated transformation. The cotyledons of tomato were used for transformation. Positive transgenic plants were screened via PCR with a CaMV35S promoter primer and a gene-specific primer. After PCR and qRT-PCR analyses, several independent homozygous transgenic lines expressing abnormal levels of *SUS3* (OE1-2, OE2-4, OE3-2, Ri1-1, Ri8-3, and Ri17-2) and WT (M82) were selected for further analysis.

### Cold stress assays

Six-week-old *SUS3-*OE lines, *SUS3-*RNAi lines, and WT were placed in a 4°C constant temperature chamber for 7 days for phenotype observation and photographing. Three replicates were set for each treatment, with 15 seedlings per replicate. The third fully expanded leaf from the apical meristem was harvested and snap-frozen in liquid nitrogen and then stored in a −80°C freezer for physiological and biochemical parameters.

### Measurements of the physiological indexes of cold stress

Malondialdehyde (MDA), relative electrolyte leakage levels, and proline were determined as previously described (Campos et al., 2003). The anthrone colorimetry method (Fukao et al., 2006) was adopted to determine the content of soluble sugar. Three biological replicates were analyzed for each treatment.

#### Superoxide radical detection

The fully expanded leaves (first from the apical meristem) from 6-week-old seedlings of *SUS3-*OE and WT were used for nitroblue tetrazolium (NBT) staining. Three replicates were set for each treatment, with 15 leaves per replicate. The NBT staining method as previously described (Xia et al., 2009) was adopted.

### Measurement of enzyme activity

Extraction of enzyme solution: A 0.2-g sample, which had been stored in a −80°C freezer, was weighed and combined with precooled phosphate buffer (pH 7.8, with 0.1 mM of EDTA) and 1.8 mL of 1% PVP. After mixing, the resulting mixture was subjected to centrifugation at 4°C and 12,000 rpm for 15 min. The supernatant obtained was considered to be the enzyme extract. The levels of superoxide dismutase (SOD), catalase (CAT), ascorbate peroxidase (APX), and peroxidase (POD) were quantified through determination as described in previous studies (Jimenez et al., 1997; Morohashi, 2002; Al-aghabary et al., 2005).

### Subcellular localization

To investigate the localization of SUS3, the full-length CDS of *SUS3* was cloned into the dual vector pGDG between *Xho*I and *Apa*I from the cDNA of A57. To fuse the target gene with the reporter gene, we introduced two additional base pairs CT after the *Xho*I restriction site. The target gene was fused with the C-terminus of a green fluorescent protein (GFP) reporter gene and expressed under the control of the 35S promoter (**Supplementary Table S2**) for tomato protoplasts. After 18 h of incubation, the image of GFP and red fluorescent protein (RFP) fluorescence was captured by confocal laser scanning microscopy (Leica SP8, Germany).

## Results

### RNA-seq analysis of extreme cold-sensitive and cold-tolerant tomato varieties

**Figure 1:**
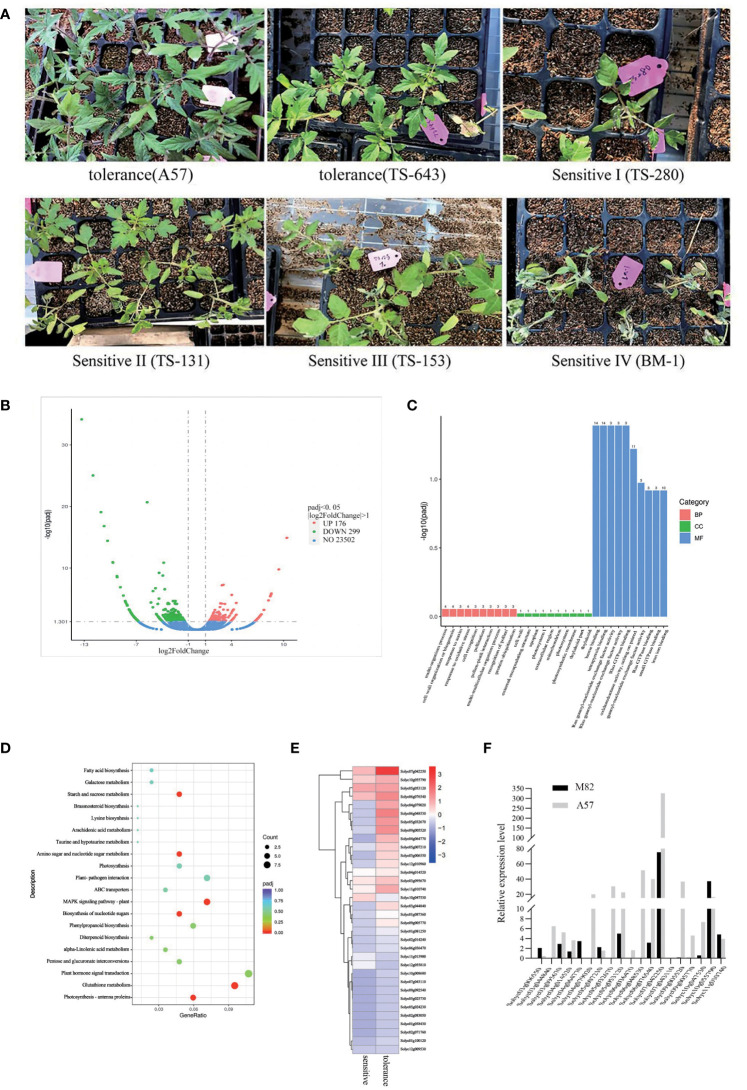
Differentially expressed genes (DEGs) in cold-sensitive and cold-tolerant tomato varieties. **(A)** Partial cold-sensitive and cold-insensitive tomato varieties. **(B)** Volcano map of DEGs. **(C, D)** Significantly enriched GO terms **(C)** and KEGG pathways **(D)** of DEGs. **(E)** Heatmap of gene expression related to sugar metabolism. **(F)** Gene expression related to sugar metabolism by qRT-PCR.

In 223 natural populations, 147 were not sensitive to cold stress and no damage was observed on their leaves, while 76 showed sensitivity to cold stress and showed varying degrees of damage. Therefore, based on the degree of damage, the impact of cold damage can be graded to different levels (**Figure 1A**). To study the effect of cold stress on whole-genome gene expression patterns, RNA-seq was performed in 20 cold-sensitive and 20 cold-tolerant accessions. There were 475 differentially expressed genes (DEGs), consisting of 176 upregulated genes and 299 downregulated genes (**Figure 1B**). The Gene Ontology (GO) terms of DEGs included heme binding, oxidoreductase activity, tetrapyrrole binding, and iron ion binding (**Figure 1C**). The Kyoto Encyclopedia of Genes and Genomes (KEGG) pathways of DEGs included starch and sucrose metabolism, biosynthesis of nucleotide sugars, amino sugar and nucleotide sugar metabolism, plant–pathogen interaction, phenylpropanoid biosynthesis, and MAPK signaling pathway (**Figure 1D**). We detected the expression of 475 DEGs, and a total of 34 significant expression (padj ≤ 0.01) were identified (**Supplementary Table S3**). Thirty-four DEGs have a higher expression level by RNA-seq (**Figure 1E**). In order to verify the accuracy of RNA-Seq, 34 DEGs were selected to quantify the expression with M82 (cold-sensitive) and A57 (cold-tolerant) by qRT-PCR (**Supplementary Table S4**). The findings were largely consistent with RNA-seq. The expression levels of *Solyc07g04255*0 were the highest (**Figure 1F**), which indicates that *SUS3* is a major candidate for having an important influence on cold tolerance (**Supplementary Table S3**).

### Molecular characterization of *SUS3*

We analyzed and predicted the structural features and function of *SUS3* at the molecular level. The length of the gene is 2,955 bp, which includes an open reading frame (ORF) of 2,418 bp, starting at position 273 with ATG and ending at position 2690 with TAA. It encodes a protein with a length of 806 amino acids and a molecular weight of 92.59 kDa (**Supplementary Figure S1A**). *SUS3* had eight helical segments that had 10–70, 100–250, 270–330, 350–450, 480–500, 550–610, 620–660, and 690–780 amino acid sequences and did not contain a signal peptide, and it was a stable non-transmembrane and hydrophilic protein (**Supplementary Figures S1B–D**).

**Figure 2:**
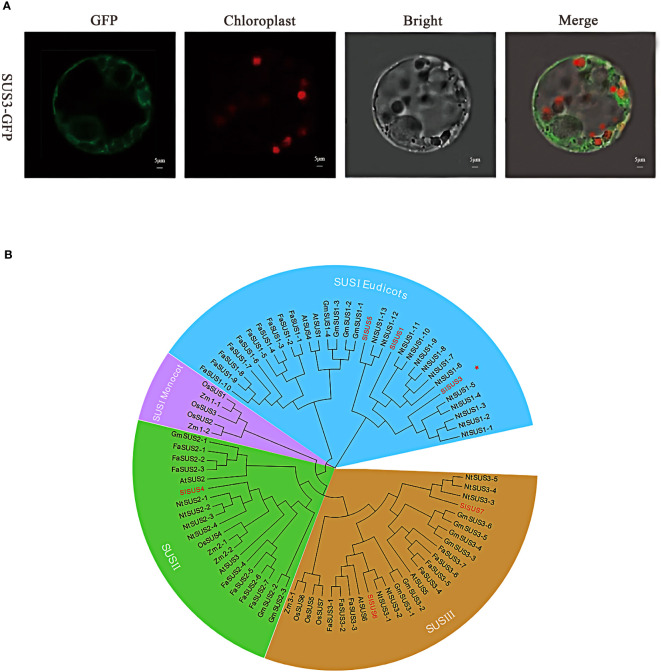
Molecular characterization of *SUS3*. **(A)** Subcellular localization of the SUS3 protein in protoplasts. **(B)** *Agrobacterium* strains carrying the GFP-SUS3 were digested into protoplasts in tobacco leaves. Bright-field, green fluorescent, red fluorescent, and merged images are shown.

In order to verify the protein function of *SUS3*, the subcellular localization of tomato protoplast confirmed that the GFP was irregularly dotted around the cells (**Figure 2A**). *SUS3* was specifically expressed in the cytoplasm and cell membrane. This result suggests that the *SUS3* is mainly located in the cytoplasm and cell membrane. To further investigate the evolutionary relationship of *SUS3* and its families, 24 family genes from strawberry (*FaSUS1-1~FaSUS1-10*, *FaSUS2-1~FaSUS2-7*, *FaSUS3-1~FaSUS3-7*), 6 genes from *Arabidopsis* (*AtSUS1~AtSUS6*), 13 genes from soybean (*GmSUS1-1~GmSUS1-4*, *GmSUS2-1~GmSUS2-3*, *GmSUS3-1~GmSUS3-6*), 7 genes (*OsSUS1~OsSUS7*) from rice, 5 genes (*ZmSUS1-1~ZmSUS2-1*, *ZmSUS2-1~ZmSUS2-2*, *ZSUSm3*) from maize, and 22 genes (*NtSUS1-1~NtSUS1-13*, *NtSUS2-1~NtSUS2-4*, *NtSUS3-3*, *NtSUS3-5*) from tobacco were analyzed. The results showed that the SUS family genes and tobacco SUS genes had high homology, implying that the SUS families and tobacco had similarities in biological functions and growth regulatory mechanisms. Meanwhile, *SUS3* has the farthest evolutionary relationship with the other five SUS family genes, and it is speculated that it may exercise different biological functions (**Figure 2B**).

### Response of *SUS3* expression to different stresses

**Figure 3:**
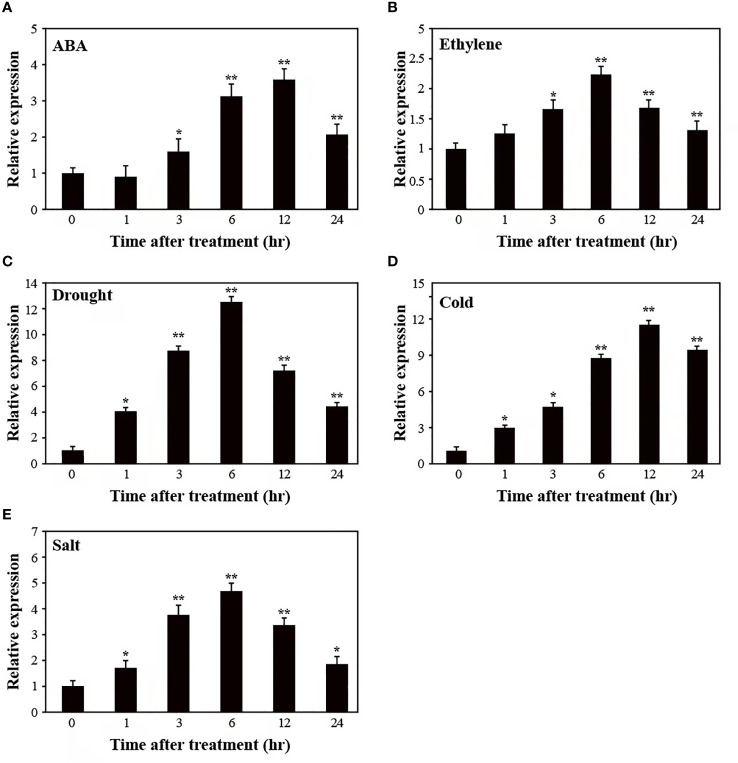
Relative expression pattern of *SUS3* under abiotic stress and hormone induction. **(A–E)** Expression pattern of *SUS3* in ABA, ethylene, drought, cold, and salt. Four-week-old seedlings were stressed with ABA (100 μM), ethylene (200 μM), drought (dehydration), cold (4°C), and salt (400 mM of NaCl) treatments for 0, 1, 3, 6, 12, and 24 h, respectively. The relative expression levels were determined using qRT-PCR. The expression levels of the appropriate controls were set to a value of 1. Data are presented as mean ± standard deviation of three biological replicates. Asterisks indicate statistically significant differences between transgenic lines and wild type. *, *p* < 0.05; **, *p* < 0.01.

To test whether *SUS3* might contribute to responses to other abiotic stresses, we examined the expression pattern of *SUS3* in AC plants after subjecting them to ABA, ethylene, drought, cold, and salt stresses (**Figure 3**). A mild induction of *SUS3* expression was detected in response to ABA, ethylene, and salt stresses, and the *SUS3* was strongly induced by drought and cold treatment. In the course of 24 h under these treatments, the expression of *SUS3* showed an initial increase followed by a decrease. More specifically, the expression level of *SUS3* continued to increase significantly within 6–12 h and reached the maximum.

### *SUS3* positively regulates the cold tolerance of tomato

**Figure 4:**
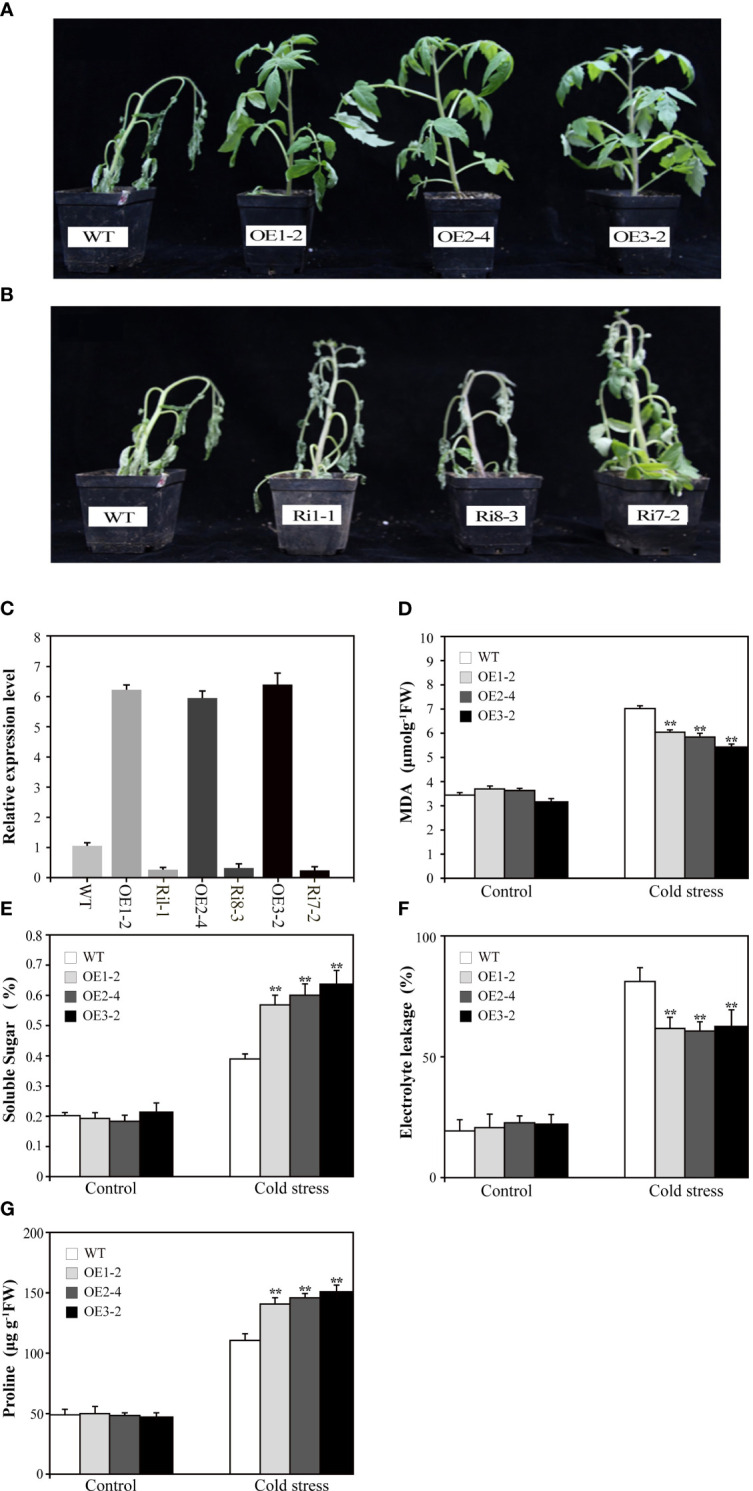
Overexpression of *SUS3* improves the cold tolerance in tomato. **(A, B)** Phenotype of transgenic seedlings expressing abnormal levels of *SUS3* and WT seedlings under cold stress conditions. The *SUS3*-OE lines (OE1-2, OE2-4, and OE3-2), *SUS3*-RNAi lines (Ri1-1, Ri8-3, and R7-2), and WT seedlings were grown at 4°C for 7 days. **(C)** Relative expression levels of *SUS3* in transgenic lines and WT. Comparison of MDA content **(D)**, soluble sugar **(E)**, relative electrolyte leakage **(F)**, and proline **(G)** in *SUS3*-OE lines and WT under cold stress and optimal conditions. Data are presented as mean ± standard deviation of three biological replicates. Asterisks indicate statistically significant differences between transgenic lines and wild type. *, *p* < 0.05; **, *p* < 0.01.

To investigate the biological functions of *SUS3*, we transformed M82 with the 35S:SUS3 construct. Three independent OE lines (OE1-2, OE2-4, OE3-2) and three RNAi lines (Ri1-1, Ri8-3, Ri17-2) were chosen for further characterization (**Figure 4C**). We tested the cold tolerance of the *SUS3* transgenic seedlings. After cold stress for 7 days, the leaves of the wild type showed wilting, and the stems became shriveled and bent and were unable to uphold upright. In contrast, the *SUS3-*OE lines in their leaves were capable of preserving regular upward growth (**Figure 4A**). However, the phenotype of the *SUS3-*RNAi lines was similar to that of WT under cold stress and did not demonstrate more wilting (**Figure 4B**). Therefore, it suggests that the loss of *SUS3*-RNAi lines might not significantly affect cold tolerance.

To evaluate cold tolerance, we analyzed the MDA, relative electrolyte leakage, and proline and soluble sugar contents between transgenic lines and WT plants under cold stress. Under optimal growth conditions, there is no difference between transgenic lines and WT in the above indexes. After 7 days of cold treatment at 4°C, MDA and relative electrolyte leakage increased in *SUS3*-OE lines and WT, but the levels were significantly lower in the *SUS3*-OE lines than in WT (**Figures 4D, F**). These results indicate that *SUS3* can alleviate the damage to cellular membranes that occurs during cold stress. Under cold stress, the contents of both proline and soluble sugar were significantly higher. Moreover, the increase of sugar in leaves of the *SUS3*-OE lines was significantly higher than in WT.

**Figure 5:**
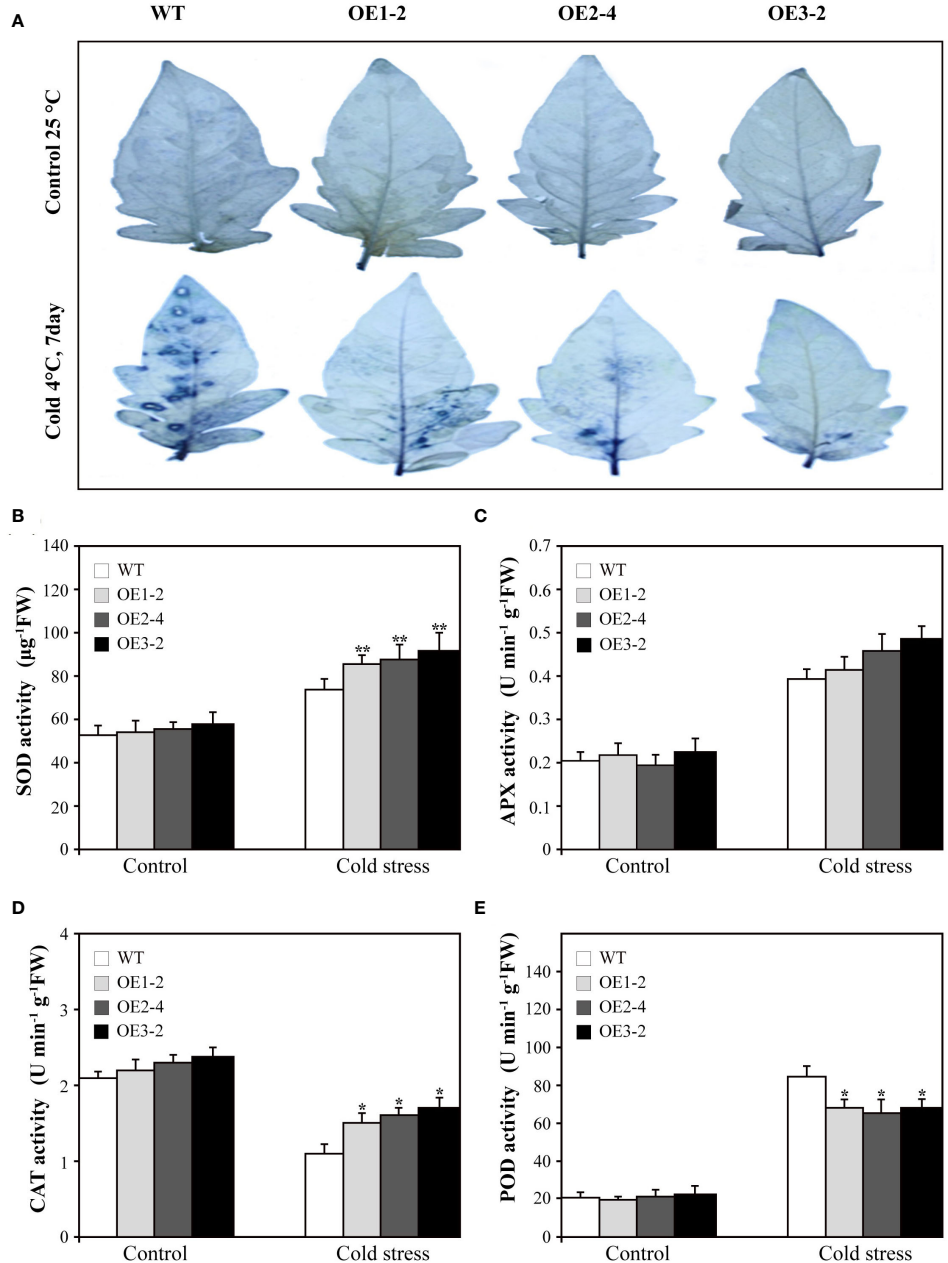
Superoxide radicals(O_2_^−.^) accumulation and related enzyme activity of *SUS3*-OE lines and WT under cold stress and optimal conditions. **(A)** NBT staining used to detect the accumulation of superoxide radicals. The *SUS3*-OE lines (OE1-2, OE2-4, and OE3-2) and WT under cold stress and optimal conditions were incubated at 25°C in the dark for 2 h. Comparison of SOD content **(B)**, APX **(C)**, CAT **(D)**, and POD **(E)** in *SUS3*-OE lines and WT grown under cold stress and optimal conditions. Data are presented as mean ± standard deviation of three biological replicates. Asterisks indicate statistically significant differences between transgenic lines and wild type. *, *p* < 0.05; **, *p* < 0.01.

Various abiotic stresses often result in the excessive accumulation of reactive oxygen species (ROS) in plants, causing substantial damage to multiple cellular structures (Gill and Tuteja, 2010). Therefore, we assessed the levels of superoxide radicals (O_2_^−.^) in *SUS3*-OE lines and WT. After treatment at 4°C for 7 days, the accumulation of O_2_^−.^ increased in both *SUS3*-OE lines and WT. However, the accumulation in *SUS3*-OE lines was significantly lower in comparison to the WT (**Figure 5A**). This indicates that overexpression of *SUS3* has the ability to prevent the accumulation of excessive O_2_^−.^ free radicals, thereby reducing the damage to plants and enhancing their resistance to cold damage.

In order to determine whether overexpression of *SUS3* can reduce the damage of plant cells under cold stress, we measured the activities of four key antioxidant enzymes (SOD, CAT, APX, and POD). The results showed that under optimal growth conditions, the activities of the four enzymes did not show significant differences between *SUS3*-OE lines and WT. However, after 7 days of cold treatment at 4°C, the activity of SOD and CAT was increased in *SUS3*-OE lines and WT, but the enzyme activity in *SUS3*-OE lines was significantly higher than in WT (**Figures 5B, D**). Under cold stress, APX activity in both *SUS3*-OE lines and WT also increased, but there was no significant difference (**Figure 5C**). The activity of POD increased in both *SUS3*-OE lines and WT, but the activity increased more significantly in the WT than in *SUS3*-OE lines (**Figure 5E**). Compared with the WT, overexpression of *SUS3* increased the activities of SOD and CAT and decreased the activities of POD under cold stress. There was an accumulation of fewer ROS in *SUS3*-OE lines, thus reducing the oxidative stress injury. This may lead to less ROS accumulation in *SUS3*-OE lines, thus reducing oxidative stress injury.

## Discussion

In recent years, transcriptomic, proteomic, and metabolomic research tools have been utilized increasingly in plants, which has been noted as a significant trend in botanical studies. Proteomic analyses of tomato fruit during cryopreservation show significant changes in biological processes including defense responses, photosynthesis, and protein degradation, such as heat stress proteins (HSPs), late embryonic developmental enriched proteins (LEAs), ATPases, and 26S proteasome subunits (Page et al., 2010; Sanchez-Bel et al., 2012). Currently, there is no report on the transcriptomics of low-temperature response in tomato seedlings, only reports on low-temperature storage in fruits, and the mechanisms of low-temperature response in seedlings and fruits are considerably different, which to some extent limits the candidate cloning and functional identification of cold-tolerance-related genes in tomato. We used the low-temperature-sensitive and cold-tolerant materials for RNA-seq to compare the expression difference after cold stress. A total of 34 genes were found to be significantly differentially expressed. *SUS3* (*Solyc07g042550*) was upregulated at a considerably higher level in cold-tolerant accessions than cold-sensitive accessions. Additionally, qRT-PCR analyses demonstrated that the expression of this gene was significantly higher in A57, a wild tomato, than in M82, a cultivated tomato. Through this, we confirmed that tomato *SUS3* is closely related to cold stress. Previous research indicated that plants are more likely to utilize SUS to promote sucrose catabolism and generate reducing sugars to maintain growth under adverse conditions (Fenando et al., 2000; Bologa et al., 2003). It has been reported that sugar beet, wheat, and cabbage SUS genes are highly expressed under conditions of low temperature (Marana et al., 1990; Hesse and Willmitzer, 1996; Sasaki et al., 2001). It is possible that SUS regulates intracellular soluble sugar content and osmotic pressure to enhance cold tolerance (Crespi et al., 1991). Previous studies have also demonstrated that *SUS1* and *SUS3* are cold-responsive genes, while *SUS1* and *SUS3* are respectively induced in response to hypoxia and drought in barley (Barrero-Sicilia et al., 2011). The study examined the effects of *SUS3* on cold tolerance and identified a range of tomato accessions that are cold-tolerant or cold-sensitive. This provides a theoretical foundation and enriches the available germplasm for breeding.

Bioinformatics analyses showed that SUS genes are a family. The tomato SUS family has six genes, and they are distributed across five chromosomes. The amino acid number for SUS varies from 805 AA to 891 AA, while the molecular weight ranges between 91.63 kDa and 100.75 kDa. The protein isoelectric point can range from 5.87 to 8.42, and the gene includes 11 to 15 exons (Bieniawska et al., 2007). *SUS3* is 2,955 bp long, with an open reading frame of 2,418 bp. *SUS3* does not contain a signal peptide and is a stable, hydrophilic, and non-transmembrane protein. Similar results were observed from the analysis of potatoes (Xu et al., 2012) and cotton (Lu et al., 2005). To further investigate the evolutionary relationships of the SUS family, genes can be classified into four major categories: SUSI eudicots, SUSI monocots, SUSII, and SUSIII. *SUS1*, *SUS3*, and *SUS4* genes are categorized as SUSI eudicots, while the *SUS4* gene falls into the SUSII category, and *SUS6* and *SUS7* belong to the SUSIII category. Notably, no *SUS* members of the monocotyledon category were found, which is consistent with previous classification results (Van Bel et al., 2017). A phylogenetic tree showed that the significant homology between the *SUS* in tomato and *NtSUS* in tobacco implies the similarity in biological functions and regulation mechanisms.

The evaluation of *SUS3* showed that it is responsive to cold, drought, salinity, ethylene, and ABA treatments. This gene could play a key role in abiotic stress perception and response. Consequently, our results suggested that *SUS3* is crucial in regulating abiotic stress and hormone response. To further evaluate the biological function of tomato *SUS3*, we obtained transgenic lines in tomato and analyzed their performance under cold stress. *SUS3*-OE lines showed less leaf wilting and better growth than wild-type plants, indicating that overexpressing *SUS3* can improve the tolerance of tomatoes to cold stress. However, the phenotype of the *SUS3*-RANi lines was similar to that of WT under cold stress and did not demonstrate more leaf wilting. Therefore, it is speculated that *SUS3* is not the sole participant in the stress resistance pathway, and other members may have a similar role (Barratt et al., 2009; Baroja et al., 2012).

When plants are subjected to cold stress, cell membrane damage occurs, which can be reflected by relative electrical conductivity and MDA levels (Baud and Graham, 2006; Nguyen et al., 2016). Under cold stress, although the relative electrical conductivity and MDA content in *SUS3-*OE lines increased due to cold damage, they were significantly lower than in WT. This indicates that *SUS3* functions to decrease adversity-induced peroxidation of the plasma membrane. Proline and soluble sugars contribute to maintaining cellular osmotic potential and membrane integrity (Morsy et al., 2007; Szabados and Savoure, 2010). Its accumulation also enhances plant tolerance to abiotic stresses (Cheng et al., 2007; Zhang and Huang, 2010). Overexpressing *SUS3* increased proline levels in response to low-temperature stress. Therefore, the *SUS3* gene improves cold tolerance in tomato plants. Interestingly, we discovered that the accumulation of O_2_^−.^ in *SUS3*-OE lines was significantly lower than that in wild-type plants when subjected to cold stress. SOD and CAT can scavenge ROS, while POD is involved in both ROS generation and ROS scavenging (Cosio and Dunand, 2009). Furthermore, the activities of SOD and CAT related to ROS were significantly higher in the *SUS3*-OE lines compared with WT. These results suggest that *SUS3* improves ROS scavenging capacity and reduces the damage of adversity-induced oxidative stress on cell membranes. Our results showed that overexpressing *SUS3* can reduce the damage to plant cells caused by cold stress and enhance the cold tolerance of plants.

In summary, we found that *SUS3* is a novel gene whose expression was induced by cold and positively regulates cold tolerance by reducing cell membrane damage. In addition, overexpressing *SUS3* increased the cold tolerance in tomato. Furthermore, our analyses of a population indicate that *SUS3* is a naturally occurring allele that is significantly enriched in cold-tolerant accessions and the *SUS3* allele is significantly distributed in cold-sensitive accessions. These data provide evidence that *SUS3* might play a role in cold tolerance. Therefore, our study demonstrates that high expression levels of *SUS3* can serve as direct targets for both genetic engineering and selection for enhanced cold tolerance in tomato. These findings provide essential information for breeding.

## Data availability statement

The original contributions presented in the study are publicly available. This data can be found here: https://www.ncbi.nlm.nih.gov/ accession number PRJNA1065726.

## Author contributions

SL: Writing – original draft. YW: Writing – original draft. YYL: Writing – review & editing. CL: Writing – review & editing. YEL: Writing – review & editing. WX: Writing – review & editing. ZY: Writing – review & editing.

## Supplementary material

Supplementary Figure 1 Bioinformatics characteristics of SUS3. **(A)** Nucleotide and amino acid sequence. **(B)** the transmembrane amino acid segment. **(C)** signal peptide (c- score: Original cut score, s- score: Signal score, y- score: General cut score). **(D)** hydrophilicity/hydrophobicity analyses.
