# Supplementary material for: Sucrose synthase gene SUS3 could enhance cold tolerance in tomato
Source: Front Plant Sci. 2024 Jan 25;14:1324401. doi: 10.3389/fpls.2023.1324401 (PMC10850397; doi:10.3389/fpls.2023.1324401)
Supplement: Supplementary Figure 1 — Bioinformatics characteristics of SUS3. (A) Nucleotide and amino acid sequence. (B) the transmembrane amino acid segment. (C) signal peptide (c- score: Original cut score, s- score: Signal score, y- score: General cut score). (D) hydrophilicity/hydrophobicity analyses. [file DataSheet_1.docx]

Supplementary Material

Sucrose synthase gene SUS3 could enhance cold tolerance in tomato

Shouming Li ^1,2^, Ying Wang^2^, Yuanyuan Liu^2^, Changhao Liu^2^, Yongen Lu^2*^, Wei Xu ^1*^, Zhibiao Ye ^2*^

*** Correspondence:**Zhibiao Ye
zbye@mail.hzau.edu.cn

Wei Xu
xuwei0412@shzu.edu.cn

Yongen Lu
luyongen@mail.hzau.edu.cn

#
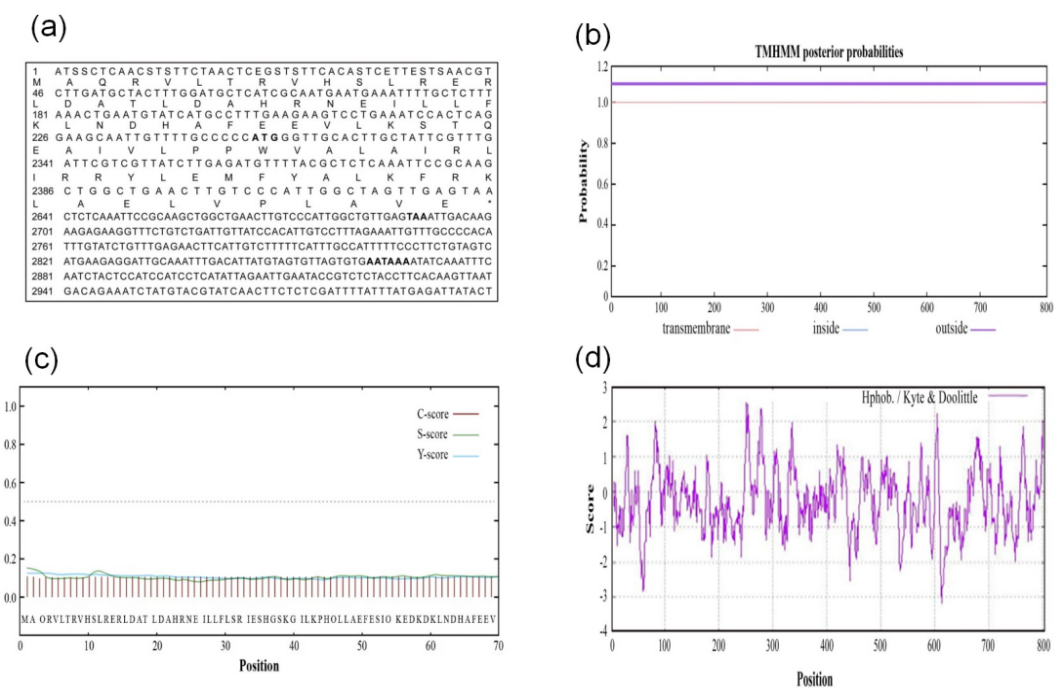
Supplementary Figures and Tables

## Supplementary Figures

**Fig. S1.** **Bioinformatics characteristics of SUS3. a** Nucleotide and amino acid sequence.**b** the transmembrane amino acid segment.**c** signal peptide (c- score: Original cut score, s- score: Signal score, y- score: General cut score).**d** hydrophilicity/hydrophobicity analyses.
