# Supplementary material for: Sucrose synthase gene SUS3 could enhance cold tolerance in tomato
Source: Front Plant Sci. 2024 Jan 25;14:1324401. doi: 10.3389/fpls.2023.1324401 (PMC10850397; doi:10.3389/fpls.2023.1324401)
Supplement: Supplementary file 3 [file DataSheet_3.docx]

**Table 2 List of primer sequences**

| **Name** | **Forward primer (5’-3’)** | **Reverse primer (5’-3’)** |
| --- | --- | --- |
| OESUS(Vector construction) | TGGAGAAAACAAGAGGAGGAAGA | TGGAGAAAACAAGAGGAGGAAGA |
| RiSUS(Vector construction) | ATGATCCATACACCAACCG | TTCTGGAAGATGACGTGAAGC |
| Actin | CATCAGGAAGGACTTGTACGG | GATGGACCTGACTCGTCATAC |
| 35S | CACCCCTTCACTTCACATTGACTTTGAG | GCAAATCCTCAGATGACTTTCA |
| SUS3(RT-PCR) | TCTTCCCAGATGAACCGCGTGAGG | CGCAGCTCATGGCCTCAACAACAG |
| SUS-OE | CATTTGGAGAGGACACGCTCGAG | TCTCATTAAAGCAGGACTCTAGA |
| SUS-Ri | GTCCAGCTGAGATCATCGTTCAT | CAGATACAAATGTGGGGCAAAC |
| SUS3-GFP | ACCTCGAGCTATGATCCATACACCAACC | ACGGTACCCCAGGCGTGAACGAAC |
| RT-PCR |  |  |
| Solyc03g006550 | CGTGGTTATGTTGCCTCAAA | ACAGCAATGTGGGAAGTGTG |
| Solyc03g044840 | AGCTCCAGTGATGAGGAGGA | TCTGCTTTCGATTCTTCCTTG |
| Solyc03g095670 | CAAGACTGAGAGCCGATTCC | CATCCCCAACTAATGCGACT |
| Solyc04g014520 | TTGAGGAGGAATGGGATGAC | GCACATCCTGGTCAAACTCA |
| Solyc04g064770 | TTCACGTATTGTGGCTGCTT | CCAGTCCCATTAGATTATCGTTG |
| Solyc04g079020 | AATGGGAGGGTTTGTTGTTG | TCGACTTCGTCAACACTGGT |
| Solyc05g007210 | GATTTGGTGGATGATGGTCA | TGGGTTGTCTTGGATTTTCTG |
| Solyc05g032670 | GGGTTCATTCCTTGGTGAAA | TTAAGCCACGATACGCTCCT |
| Solyc05g053120 | GGTCCCTCGGCCTATACTTC | CCTCGTTCCTGTGCTTCTTC |
| Solyc06g036470 | GAAGCAACCATGAGGAAAGC | GTGGACATGGATTGCCTTTT |
| Solyc06g048530 | CCCTGGAAGGAATGTGATGT | CCCAAAGGATGAGAGCAAAG |
| Solyc06g076540 | CAATTGCCCTAACGTTGTTG | AGCATCAGTAGCAGCAGCAG |
| Solyc07g042250 | GAGGCAGAAGGTGGTGTAGG | TGCCAACTTTTTCTCCATCA |
| Solyc07g043110 | CACGAGGATCGAGAAAATGTT | AATGCTCTTTGCAGCTCCTC |
| Solyc09g005520 | TCAAACCCTTGCTTGAGAATC | TGCTTTGTTCCAACTCATCG |
| Solyc09g005770 | TTCCGTCATTCACCGTTTC | TCATCCTCGAGAACAAAGCA |
| Solyc10g047530 | GGCCCTGACCGTGTTAAGTA | CTGAAAGTCCAGCGGTATCC |
| Solyc10g055790 | CCAAGCCTGTCTCTTCTGGT | GTGTCCCATCCGTAGTCACC |
| Solyc11g010740 | AATGTGGGAAGGGTGAAGTG | AAATGGAAGCCCAGTTCC |
|  |  |  |
